# 上皮细胞间质化与肿瘤的转移

**DOI:** 10.3779/j.issn.1009-3419.2011.07.11

**Published:** 2011-07-20

**Authors:** 志浩 吴

**Affiliations:** 1 300052 天津，天津市肺癌转移与肿瘤微环境重点实验室，天津市肺癌研究所，天津医科大学总医院 Tianjin Key Laboratory of Lung Cancer Metastasis and Tumor Microenvironment, Tianjin Lung Cancer Institute, Tianjin Medical University General Hospital, Tianjin 300052, China; 2 300052 天津，天津医科大学总医院放疗科 Department of Radiation Oncology, Tianjin Medical University General Hospital, Tianjin 300052, China

**Keywords:** 上皮细胞间质化, 肿瘤侵润, 转移, Epithelial-mesenchymal transition, Tumor invasion, Metastasis

## Abstract

恶性肿瘤的转移是指肿瘤从原发部位转移到远隔器官，是恶性肿瘤患者致死的常见原因。肿瘤细胞侵润周围组织，侵润血管，在转移部位侵出血管并形成新的转移灶，这一过程需要很多的分子机制参与。上皮细胞间质化在胚胎发育以及成人的损伤修复、组织再生、器官纤维化以及肿瘤进展等方面发挥重要作用。上皮细胞间质化是恶性肿瘤侵润转移的首要步骤。随着我们对于上皮细胞间质化分子机制了解的深入，使得我们更好地知晓肿瘤及其进展的过程，并为恶性肿瘤的治疗干预提供有效的方法。

## Abstract (en)

Metastasis is the transfer of malignant tumors from one organ to a distant organ. It is the most common cause of death in cancer patients. Different molecular mechanisms enable tumor cells to infltrate the surrounding tissue, invade blood vessels and leave the blood stream at a different site. Epithelial-mesenchymal transition (EMT) is critical for appropriate embryonic development, and this process is re-engaged in adults during wound healing, tissue regeneration, organ fbrosis, and cancer progression. EMT is the frst step in tumor invasion and metastasis. A detailed knowledge of the molecular requirements for EMT in human cancer will help us to beter understand tumor progression and to delineate more effective strategies for future therapeutic intervention.

恶性肿瘤的转移是恶性肿瘤患者死亡以及治疗失败的常见原因，肿瘤细胞侵润周围组织，诱导新生血管形成，侵润血管，进入血液循环并存活下来，在转移部位再侵出血管并形成新的转移灶，这一过程非常复杂，受到肿瘤微环境的影响，许多分子机制参与其中^[1]^。上皮细胞间质化（epithelial-mesenchymal transition, EMT）在生理状况下与胚胎发育有关^[2]^，近些年其在肿瘤转移方面的作用及其相关机制的研究，逐渐受到大家的重视，成为有关肿瘤转移研究方面的热点之一^[3]^。

## 1 EMT概念及分类

人体的组织主要包括两种细胞类型：上皮细胞和间质细胞，两者在细胞表型以及细胞功能上明显不同。上皮细胞通过紧密连接、粘附连接、桥粒和缝隙连接彼此相互连接，具有极性；相反间质细胞没有极性，缺少细胞间连接，能够通过细胞外基质游走^[4]^。

上皮细胞表型和间质细胞表型在某些情况下可以相互之间转化。Greenburg^[5]^于1982年首次提出了EMT的概念。EMT就是上皮细胞在一些生理或病理因素的作用下，失去细胞极性，丢失细胞间紧密连接和粘附连接，变成了具有间质细胞形态和特性的细胞，从而获得了浸润性和游走迁移的能力^[6, 7]^，这种改变被定义为EMT。

EMT根据不同的环境背景可分为三种类型：Ⅰ型主要发生在胚胎发育过程中，参与原肠胚（EMT发生时原始的上皮细胞形成间质细胞）以及神经嵴（早期的神经上皮细胞形成神经嵴细胞）的形成；Ⅱ型主要发生在器官纤维化以及炎症过程中，与器官的纤维化以及炎症的修复有关（上皮或内皮细胞形成成纤维细胞）；Ⅲ型则与肿瘤的侵润和转移有关。原发肿瘤的上皮细胞转化成间质细胞，获得了运动的能力，脱离原发部位侵袭周围组织进入血液循环，在远隔部位通过间质细胞上皮化（mesenchymal-epithelial transition, MET）形成新的转移灶^[6, 8, 9]^。

## 2 EMT与恶性肿瘤侵润和转移的关系

在人体的恶性肿瘤中90%以上是上皮细胞性肿瘤。由于EMT是上皮细胞获得迁移能力的有效方式，它成为上皮细胞癌浸润转移的一个重要途径，研究^[10]^表明它与上皮细胞恶性肿瘤的侵润和转移关系密切，受到了越来越大的关注。

肿瘤的转移是一个复杂而连续的过程，包括肿瘤细胞在原发部位的生长、侵及周围正常组织、穿透基底膜、侵及脉管进入循环系统并存活下来到达远隔部位，侵出脉管并在新的部位形成转移灶^[11]^。

有研究显示在原发肿瘤局部侵润的边缘有EMT特定基因的表达，显示出EMT可能是肿瘤侵出基底膜侵润周围正常组织的必备条件^[12, 13]^，而诱导肿瘤细胞发生EMT还能增加其穿过血管内皮细胞进入血液循环的能力^[11, 14]^。这些都显示EMT与肿瘤转移的早期阶段密切相关。此外还有研究把诱导EMT特定基因（twist）过表达的肿瘤细胞通过鼠尾静脉注射，增加了肿瘤细胞肺转移的能力，显示出EMT可能与肿瘤细胞的血管外侵有关系。当肿瘤细胞到达转移部位通过MET增殖形成与原发肿瘤类型相同的新的转移灶^[15]^。

此外，还有研究^[16-18]^显示EMT与肿瘤细胞对于表皮生长因子受体（epidermal growth factor receptor, EGFR）酪氨酸激酶抑制剂（吉非替尼、厄洛替尼）的敏感性有关，发生EMT的肿瘤细胞对于EGFR酪氨酸激酶抑制剂有抗药性。

## 3 EMT判定标准及标志物

肿瘤细胞发生EMT时除了在细胞形态、细胞极性、侵袭游走能力、抗凋亡能力等形态学和生物学行为的变化之外，还同时伴有细胞表面蛋白、细胞骨架蛋白、细胞外基质蛋白以及某些转录因子表达的异常^[9]^。如何判断肿瘤细胞是否发生了EMT，需要综合上述多种因素。体外实验判断的标准目前较为一致观点认为，符合下述情况可以判定细胞发生了EMT：细胞由多角形上皮细胞形态转变成纺锤形的间质细胞形态，失去细胞极性；迁移和侵袭能力增加；抗凋亡能力增加；诱导EMT的因素移除后仍然能够维持表型的稳定；一些上皮细胞标志物（如上皮性-钙粘蛋白、细胞角蛋白、紧密连接蛋白ZO-1）表达的降低或缺失，同时一些间质细胞标志物（成纤维细胞特异性蛋白1、波形蛋白、Ⅰ型胶原、纤粘连蛋白）的出现和表达的升高；一些分子的移位和转化（如β-连环蛋白由细胞核外转移至核内^[19]^；上皮性-钙粘蛋白转化成神经性-钙粘蛋白^[20, 21]^）以及某些转录因子（Snail、Slug或Twist^[22-24]^）表达的升高。

体内肿瘤细胞EMT的判定是一个难题，原因是理论上认为发生EMT的肿瘤细胞只占原发肿瘤的很少一部分。一些基因、转录、蛋白水平的改变往往为原发肿瘤所掩盖，较难确认和捕捉到已经发生转化的上皮性肿瘤细胞。最为直接的证据就是存在新形成的成纤维细胞以及基底膜的降解^[25]^。一些EMT的标志物可以参考体外实验，表 1列举了一些EMT的分子标志物。

**1:** EMT常见的一些标志物 Common biomarkers of EMT

名称	表达的改变	作用
细胞表面蛋白		
上皮性钙粘蛋白（E-cadherin）	↓	上皮细胞标志物，维持上皮细胞表型和极性的重要分子
神经性钙粘附蛋白（N-cadherin）	↑	分布于神经和肌肉组织中以及恶性非癌性肿瘤，N-cadherin可促进肿瘤浸润转移，EMT发生时常伴有E-cadherin向N-cadherin的转化
紧密连接蛋白（ZO-1）	↓	参与调节细胞物质转运和维持上皮极性，而且还与细胞增殖分化、肿瘤细胞转移、基因转录等过程的信息传递和调控有关
细胞骨架标志物		
*α*-平滑肌肌动蛋白（*α*-SMA）	↑	广泛分布于几乎所有的肌型细胞中，恶性肿瘤中能够反映新生血管情况，表达的增高与肿瘤的侵润和转移有关
波形蛋白（Vimentin）	↑	间质细胞标志物，表达增高使细胞的迁移能力增加并常常伴有E-cadherin表达的降低
*β*-连环蛋白（*β*-catenin）	↑	与E-cadherin和*α*-catenin连接，形成粘附连接，磷酸化后转入核内，与某些转录因子作用诱导EMT的发生
细胞角蛋白（Cytokeratin）	↓	上皮细胞标志物，具有维持正常细胞形态和功能的重要作用
细胞外基质蛋白		
纤粘连蛋白（Fibronectin）	↑	EMT发生时增高，非特异性的
转录因子		
Snail	↑	与E-cadherin启动子的E盒结合抑制其表达
Slug	↑	Snail2，形成锌指结构，能够下调*E-cadherin*基因的表达，入核的*β*-catenin可以激活*slug*基因的表达
Twist	↑	新近发现的一种诱导EMT的转录因子，能够独立地下调E-cadherin，并上调Fibronectin和N-cadherin

### 3.1 E-cadherin

上皮性-钙粘蛋白，人类的E-cadherin编码基因定位于16号染色体q22.1附近，分子量为120 kDa。分子中存在一个疏水基因，位于跨膜区，氨基末端位于细胞膜外，是钙离子的结合位点，对钙离子有高度敏感性，羟基末端位于细胞浆内。E-cadherin形成二聚体，胞外部分相互连接形成粘附连接，细胞内部分与β-连环蛋白（β-catenin）或γ-连环蛋白（γ-catenin）结合，β-catenin通过与α-连环蛋白（α-catenin）结合后与肌动蛋白丝结合，而γ-catenin可以直接与肌动蛋白丝结合。E-cadherin/catenin复合物在维持上皮细胞表型和粘附连接中具有重要作用^[26, 27]^。许多研究^[26, 28-30]^显示E-cadherin/catenin复合物的降解，或者E-cadherin表达的降低或缺失与肿瘤细胞的分化、肿瘤的分期、侵润、转移以及预后密切相关。E-cadherin是EMT最为重要的标志物之一^[31]^。

### 3.2 Fibronectin

纤粘连蛋白，是高相对分子质量的粘附性的糖蛋白。纤粘连蛋白以可溶的形式存在于血浆和各种体液中，称为血浆纤维粘连蛋白；以不溶的纤维存在于细胞外基质、细胞之间及某些细胞表面，称为细胞纤维粘连蛋白。由两个亚基组成的二聚体，每个亚基的相对分子质量约250 kDa，各亚基在C末端形成两个二硫键交联。EMT发生时伴有Fibronectin的增高，但是非特异性的，因为很多细胞（如成纤维细胞、单核细胞、上皮细胞）都可以产生Fibronectin^[32, 33]^。

### 3.3 Snail

其基因产物结合到E-cadherin启动子的E盒，从而抑制其表达^[34]^。在一些上皮性肿瘤细胞中是EMT的诱导因子。当上皮细胞获得间质细胞表型时，snail蛋白表达增高并结合到E-cadherin启动子的E盒并抑制其活性。EMT的许多信号传导通路都是通过snail发挥作用^[35]^。Snail的表达增高与肿瘤的侵润转移和预后也存在密切联系^[36, 37]^。近年来slug^[38]^、twist、CBF-A和KAP-1等转录因子也逐渐受到大家的重视^[39]^。

EMT的发生机制十分复杂，不同的肿瘤存在不同的调控机制，而同一肿瘤细胞EMT的调控也可能存在多个途径。EMT的信号传导途径是一个复杂而庞大的网络。有关EMT对于乳腺癌、肺癌、直肠癌、肝癌、前列腺癌转移影响的研究^[33, 40]^显示TGF-β/Smad、RTK/RAS、Wint/β-catenin等均能够诱导EMT在不同肿瘤中的发生。近期的研究显示NF-kB^[41]^、GSK-3β、STAT3/LIV-1等信号途径也会诱导EMT的发生^[42]^。不同的信号传导通路之间也存在相互关联。

## 4 问题与展望

多数有关EMT与肿瘤侵润、转移相关性的研究仅仅局限于体外实验以及少数的动物实验，至今仍存在很多的争议之处。原因是在体内发生侵润转移的肿瘤细胞只占原发肿瘤的很少一部分，当它们发生EMT时的一些基因、转录、蛋白水平的改变往往为原发肿瘤所掩盖。如何确认和捕捉到已经发生转化的上皮性肿瘤细胞是一个难题^[43]^。新的显像技术以及细胞标记方法为问题的解决带来了希望。目前理论上认为肿瘤细胞的转移需要经过EMT和MET两个过程。两者的发生是一个动态连续的过程，受到肿瘤微环境的影响。相关的分子机制、动物模型、分子探针以及人体研究将来有望成为肿瘤研究领域的热点，为肿瘤转移早期的诊断、预后的评估提供有价值的信息，并且有望为肿瘤的治疗提供新的靶点^[44-46]^。
